# Alterations in the proteomic composition of *Serratia marcescens* in response to manganese (II)

**DOI:** 10.1186/s12896-018-0493-3

**Published:** 2018-12-29

**Authors:** Pollyana Santos Queiroz, France Anne Dias Ruas, Natália Rocha Barboza, William de Castro Borges, Renata Guerra-Sá

**Affiliations:** 1 Laboratório de Bioquímica e Biologia Molecular, Departamento de Ciências Biológicas (DECBI) & Instituto de Ciências Exatas e Biológica (NUPEB), Campus Morro do Cruzeiro, Universidade Federal de Ouro Preto, Ouro Preto, MG Brazil; 2 Laboratório de Enzimologia e Proteômica, Departamento de Ciências Biológicas (DECBI), Núcleo de Pesquisas em Ciências Biológicas, Universidade Federal de Ouro Preto, Ouro Preto, Minas Gerais Brazil

**Keywords:** Mn (II)-modulated protein expression, Mn (II) oxidation, Multicopper oxidase, *Serratia marcescens* proteome

## Abstract

**Background:**

Proteomics is an important tool for the investigation of dynamic physiological responses of microbes under heavy metal stress. To gain insight into how bacteria respond to manganese (II) and identify the proteins involved in Mn (II) oxidation, the shotgun proteomics approach was applied to a potential Mn (II)-oxidizing *Serratia marcescens* strain cultivated in the absence and presence of Mn (II).

**Results:**

The LG1 strain, which grew equally well in the two conditions, was found to express a set of proteins related to cellular processes vital for survival, as well as proteins involved in adaptation and tolerance to Mn (II). The multicopper oxidase CueO was identified, indicating its probable participation in the Mn (II) bio-oxidation; however, its expression was not modulated by the presence of Mn (II). A set of proteins related to cell and metabolic processes vital to the cells were downregulated in the presence of Mn (II), while cell membrane-related proteins involved in the maintenance of cell integrity and survival under stress were upregulated under this condition.

**Conclusions:**

These findings indicate that the LG1 strain may be applied successfully in the bioremediation of Mn (II), and the shotgun approach provides an efficient means for obtaining the total proteome of this species.

## Background

Manganese (Mn) contamination of industrial waters, groundwater and drinking water is a growing problem in many parts of the world [1]. Excess soluble Mn (II) may be toxic and cause great damage to health; therefore, the development of methods for its removal from contaminated environments is critical [2, 3]. Mn (II)-oxidizing bacteria exhibit tolerance to this metal and oxidize it to insoluble Mn (III)/(IV) oxides, promoting their precipitation. Various mechanisms operate in Mn removal, ranging from indirect pathways such as biosorption to direct pathways involving enzymes such as multicopper oxidases (MCO), hemeperoxidases and a two-component regulatory protein [4].

The molecular mechanisms involved in Mn (II) removal as well as the physiological responses of the bacteria in response to the presence of this metal can be inferred via proteomic analysis. Proteomic studies of environmental microorganisms have been conducted because of the important attributes of these species that allow them to tolerate, degrade or precipitate toxic compounds, as well as their versatility in using electron donors, electron acceptors or sources of carbon and energy [5]. The application of proteomic analysis allows us to investigate microbial characteristics through the expression of proteins, and also provides an overview of the protein complement of these biological systems [5, 6].

Among the few proteomics studies involving Mn (II)-oxidizing bacteria, the comparative analysis of the proteome of two marine bacteria, a Mn (II) oxidant *Roseobacter* sp. AzwK-3b, with the non-oxidant *Ruegeria* sp. TM1040 can be highlighted. Both bacteria express a variety of proteins in response to Mn; however, few proteins were found to be expressed in relation to the oxidation of Mn (II). The hemeperoxidase enzyme was identified in the proteome of the oxidant strain, but its expression was not modulated by the presence of Mn (II) [7].

*Serratia marcescens* strains can tolerate and remove high concentrations of Mn (II) [8]. However, its proteomic profile in response to this metal remains to be elucidated. This species is an opportunistic pathogen belonging to the Enterobacteriaceae family characterized by its ability to produce important pigments and secrete enzymes such as phospholipases, proteases and nucleases [9, 10]. The few proteomic studies involving *S. marcescens* have only identified antibacterial toxins secreted by the type VI secretion system [11], proteins of chitinolytic machinery [12], proteins in response to stress caused by exposure to excess radio waves [13], proteins modulated by microgravity [14] and proteins expressed in autotrophic and heterotrophic conditions [15].

In order to identify the proteins involved in the response of *S. marcescens* to the presence of Mn (II) and involved in oxidation, the main objective of this study was to obtain the proteomic profile of a Mn (II)-oxidizing *S. marcescens* LG1 strain. The LG1strain was grown in the absence and presence of Mn (II), and its total proteome was obtained through the shotgun proteomic approach, which is applied for the first time to the study of Mn (II)-oxidant *S. marcescens* strain in this present work.

## Results

### Mn (II) bio-oxidation by LG1 strain

**Fig. 1:**
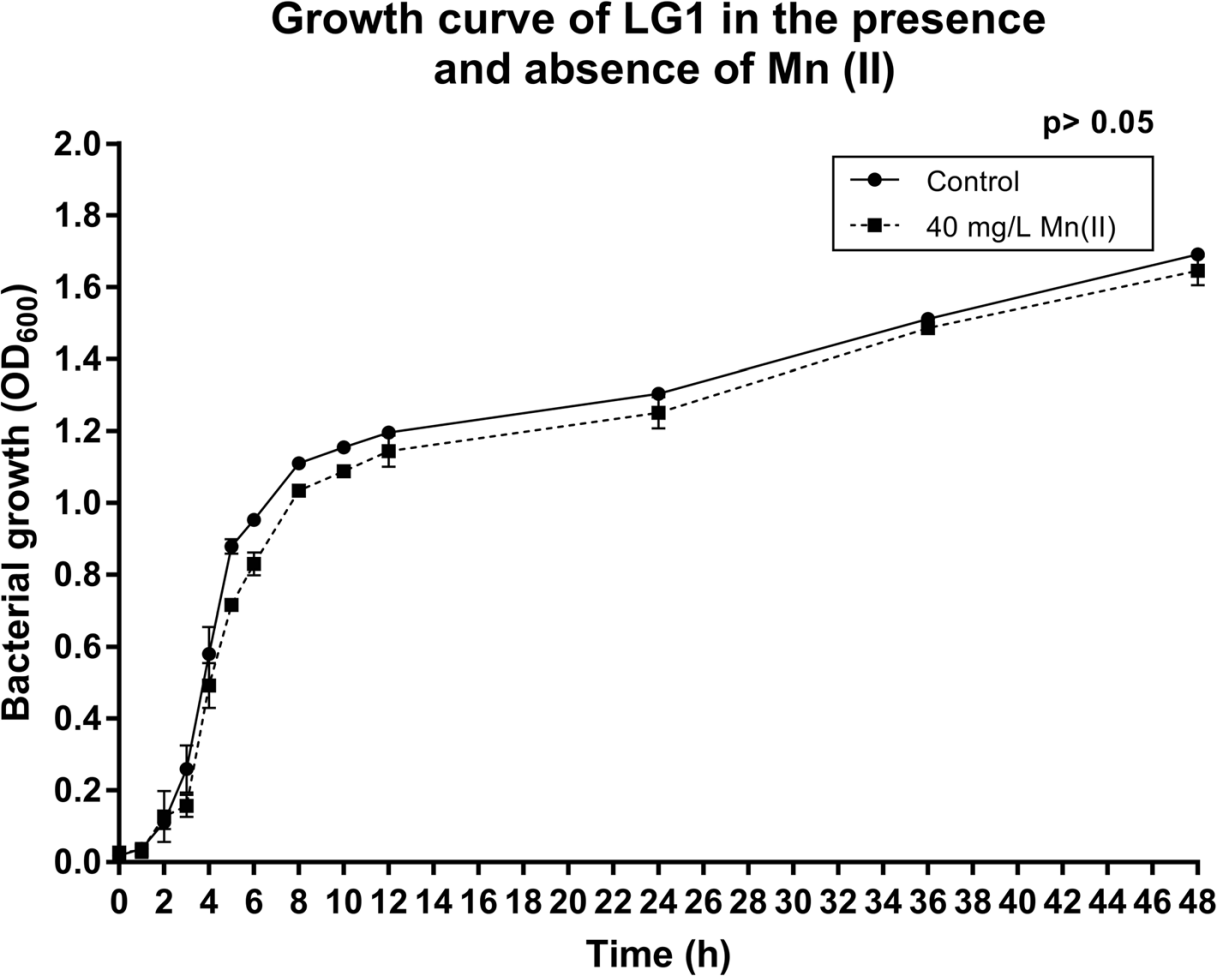
LG1 growth in the absence and presence of high concentrations of Mn (II) (40 mg/L) for 48 h. LG1 grew equally well under both conditions, demonstrating tolerance to Mn (II)

**Fig. 2:**
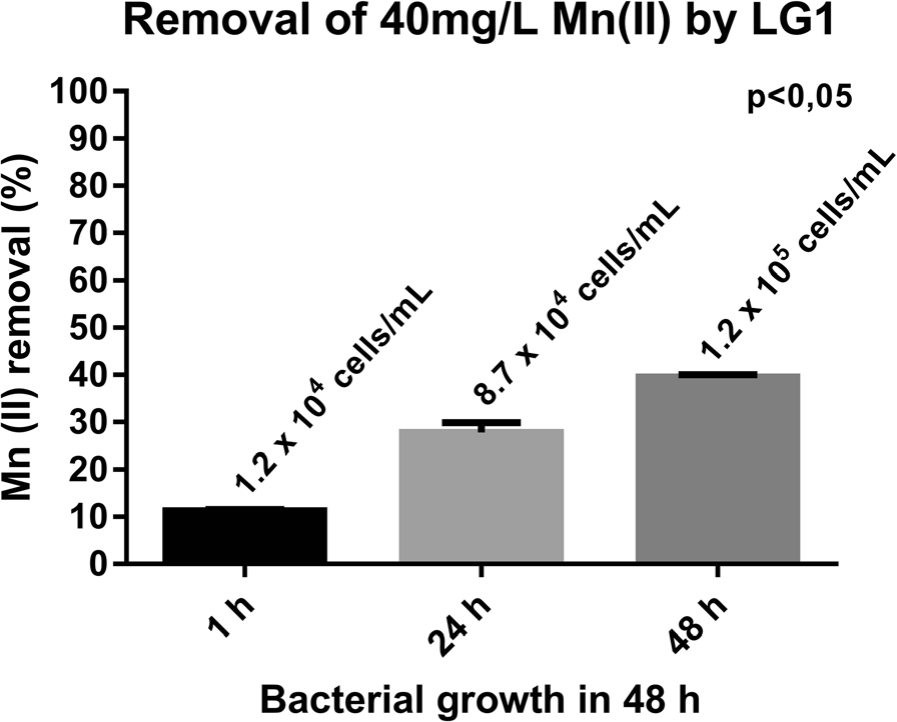
Mn (II) removal by LG1 grown in 48 h. As the number of cells increased, the removal rate of Mn (II) also increased

The LG1 strain exhibited high growth, both in the presence and absence of 40 mg/L of Mn (II) and no significant difference was found between the curves for each experiment (*p* > 0.05) (Fig. 1). A progressive growth under each condition was observed up to 12 h and thereafter, a slowdown in growth was noted. The rate of Mn (II) removal increased with bacterial growth (*p* < 0.05) (Fig. 2) and the LG1 strain removed 39.5% of Mn (II) present in the medium at 48 h. In addition, LBB indicated the presence of oxidized species of Mn at 48 h.

### Proteins identified in the total proteome in the absence and presence of Mn (II): MCO enzyme identified

**Fig. 3:**
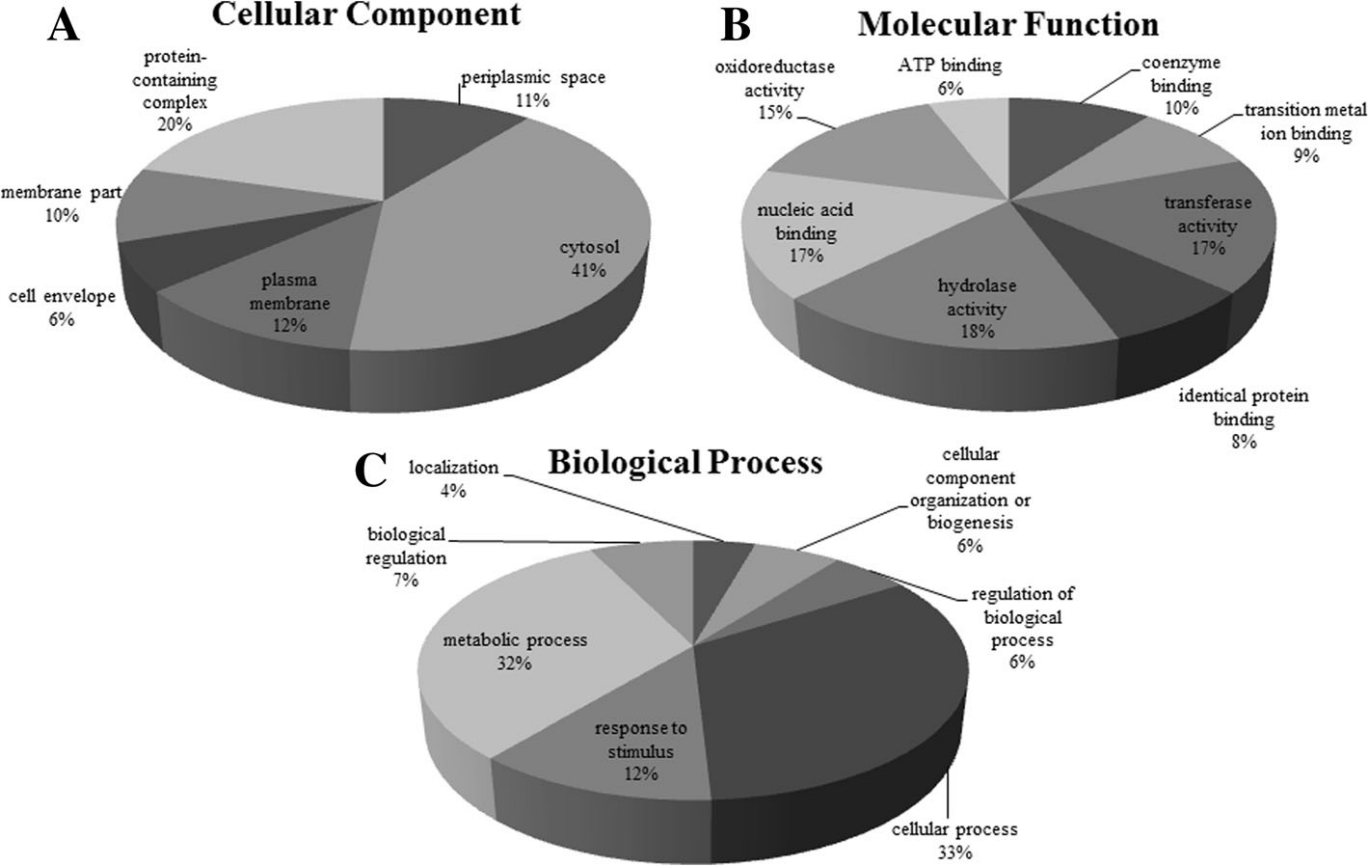
Categorization of the LG1 total proteins by Blast2GO by cellular component (**a**), molecular function (**b**) and biological process (**c**)

A total of 1577 proteins were identified in the three biological replicates of the LG1 strain for a single experiment. Among these, 787 proteins were identified in the absence of Mn (II), 52 in the presence of Mn (II) and 738 in both samples. The classification of these proteins by UniProtKB and Blast2GO indicated that most of them were present in the cytosol (41%) in the form of protein complexes (20%) and in the plasma membrane (12%) according to the cellular components (Fig. 3a). The main molecular functions of these proteins were as follows: hydrolase activity (18%), transferase activity (17%), and nucleic acid binding (17%) (Fig. 3b). In relation to the biological processes, most of the proteins were involved in cellular processes (33%), metabolic processes (32%) and response to stimulus (12%) (Fig. 3c). More specifically, the proteins were found to play roles in carbohydrate biosynthesis; metabolism of carbon, amino acids, fatty acids and ribosomal proteins; nucleotide biosynthesis; transcription; translation; cell division; DNA replication; vitamins synthesis; virulence factors and oxidoreductase activity.

For both conditions, in the absence and presence of Mn (II), proteins present in cell components related to bacterial tolerance to heavy metals, such as lipoproteins from the lipopolysaccharide (LPS) assembly and peptidoglycans, glycoproteins that are part of extracellular polymeric substances (EPS), and other proteins possessing this function and antioxidant activity such as cytochromes, glutathione, glutaredoxin, thioredoxin, catalase-peroxidase and superoxide dismutase, were identified. The MCO was additionally identified. This enzyme, which participates in oxidoreduction processes, was found both in the cellular envelope and in the periplasmic space. According to Blastp data, MCO was confirmed with 100% identity with the MCO CueO, whose role in Mn (II) removal processes has previously been reported [16].

### Differentially expressed proteins in both groups

**Fig. 4:**
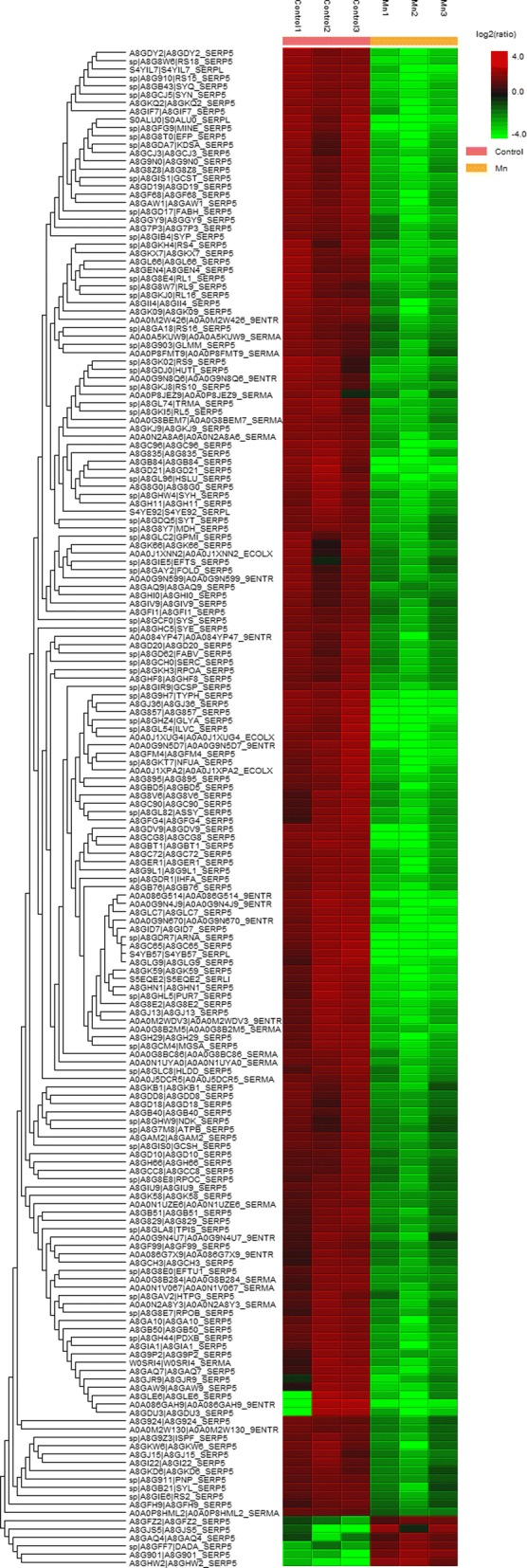
Heatmap of LG1 proteins upregulated and downregulated in the absence (control) and presence of Mn (II). Darker shades indicate higher expression and lighter shades represent lower expression of the proteins

Results showed that 182 proteins were overexpressed, with 176 being more abundant in absence of Mn (II) while six proteins were more abundant in the presence of Mn (II) (Fig. 4). In the absence of Mn (II), proteins related to the metabolism of carbohydrates (malate dehydrogenase), carbon (transketolase), amino acids and nucleotides (serine-tRNA ligase), fatty acids (3-oxoacyl-[acyl-carrier protein] synthase III), vitamins synthesis (riboflavin biosynthesis) were identified; further, proteins involved in transcription (DNA-directed RNA polymerase), translation (elongation factor), cell motility (flagellin), adhesion (pili assembly), cell division (cell division topological specificity factor) and ribosomal proteins (30/50S ribosomal protein) were found.

In general, the proteins identified in the presence of Mn (II) were related to processes involving the plasma membrane. The outer membrane protein BamB, ATP-dependent zinc metalloprotease FtsH, D-amino acid dehydrogenase, ubiquinol oxidase subunit II, dihydroxyacetone kinase, bifunctional protein PutA were the most differentially expressed under this condition (Table 1).

**Table 1:** Proteins identified as upregulated in the presence of Mn (II) and information on Uniprot accession, protein name, gene, function, subcellular location and mass (Da)

UniProt Accession	Protein name	Gene	Function	Subcellular location	Mass (Da)
A8GHW2_SERP5 m	Outer membrane protein assembly factor BamB	bamB	Part of the outer membrane protein assembly complex	Cell outer membrane	42113
A8G901_SERP5	ATP-dependent zinc metalloprotease FtsH	ftsH	Plays a role in the quality control of integral membrane proteins	Cell inner membrane	70508
DADA_SERP5	D-amino acid dehydrogenase	dadA	Oxidative deamination of D-amino acids.	–	47212
A8GAQ4_SERP5 m	Ubiquinol oxidase subunit2	Spro_1090	Copper ion binding cytochrome-c oxidase activity	Cell membrane	34933
A8GJS5_SERP5	Dihydroxyacetone kinase, L subunit	Spro_4271	Glycerone kinase activity	–	22825
A8GFZ2_SERP5	Bifunctional protein PutA	Spro_2931	Oxidizes proline to glutamate for use as a carbon and nitrogen source	–	144402

## Discussion

In this study, we showed for the first time the total proteome of the Mn (II)-oxidizing *S. marcescens* LG1 strain, in two different conditions: the absence and presence of Mn (II) ion. The analysis allowed us a better physiological understanding of the tolerance and oxidation of Mn by the LG1 strain of this species. *Serratia* species isolated from different environments exhibit the ability to remove heavy metals and radioactive elements, both by enzymatic pathways and indirect mechanisms such as biosorption [8, 13, 17–21]. As expected, the Mn (II) - resistant LG1 strain showed a good Mn (II) removal rate even during the short period of growth observed, and was able to produce oxidized species of Mn, confirming its potential for bio-oxidation of this heavy metal. Few studies have performed analysis of the proteomes of these species in order to elucidate the molecular mechanisms underlying the resistance to, and removal of, these toxic contaminants. Only Zakeri et al. (2012) reported the analysis of the differentially expressed proteome of a radium-biosorbent *S. marcescens* strain in response to radioactive stress. The authors used two-dimensional polyacrylamide gel electrophoresis and mass spectrometry to identify the proteins involved [13]. The shotgun bottom-up proteomic approach used in the present study allowed us to identify not only the protein (s) that may be involved in Mn (II) tolerance and bio-oxidation, but also to obtain the first total proteome of a *Serratia* species, the strain *S. marcescens* LG1.

In general, the proteins identified in the absence and presence of Mn (II) were structural proteins and enzymes related to major cellular and metabolic processes required for growth and survival of gram-negative bacteria. Most of these proteins were identified mainly in the absence of Mn (II) and between both analyzed conditions; this shows that the presence of high concentrations of Mn (II) caused little interference in the set of expressed proteins and demonstrates the intrinsic adaptability of the LG1 strain to Mn, once the strain growing equally well in the absence and presence of Mn (II). Proteins from structures related to heavy metal tolerance may have contributed to their good adaptability. EPS, LPS and peptidoglycans are bacterial structures that protect them from stress caused by excessive metals in the environment [22–25]. *S. marcescens* produces EPS that, besides containing carbohydrates, comprises large amounts of enzymes and structural proteins such as glycoproteins [26, 27]. These enzymes and proteins maintain the cell’s structural integrity and stability for signaling and inter-cellular communication [28]. LPS, which is composed of lipids and polysaccharides, and which may contain associated lipoproteins, enables gram-negative bacteria to resist high levels of metals in their environment [24]. Soluble cytochromes, glutathione, glutaredoxin and thioredoxin systems are also important antioxidants and determinants in tolerance to some heavy metals for some bacteria [29–32]. Learman and Hansel (2014), when analyzing the proteome of bacteria of the *Roseobacter* clade in the absence and presence of Mn (II), also found proteins with antioxidant activity (superoxide dismutase, reductases, and several different catalases and peroxidases), and these proteins were not strongly influenced by the presence of Mn(II) [7]. These data indicate that regardless of the presence of this metal, antioxidant proteins play an important role in the survival of these species.

The identification of the MCO CueO enzyme in the present work corroborates the potential of the LG1 strain for oxidizing Mn (II). The major pathway for bacterial oxidation of Mn (II) is enzymatic, and, generally, an MCO-like enzyme is required to oxidize Mn (II) [4]. CueO is a periplasmic MCO that is associated with the copper efflux system [33]. Its ability to oxidize Mn (II) and to produce Mn oxides was demonstrated by Su et al. (2014), who cloned the gene for this enzyme from *Escherichia coli* and examined the Mn (II) oxidative activity with purified CueO (in vitro) and a recombinant strain (in vivo) [16]. Virtually all MCO consist of three domains of cupredoxin [4], and based on the similar sequence of MCO CueO available in the NCBI, this enzyme also possesses these domains. However, the presence of a MCO corroborated with our previous study, which showed the production of Mn oxide by LG1 strains and Mn oxidized species inside of LG1 cell by electron energy loss spectroscopy analyses (EELS) [34]. In addition, we also proved Mn oxide production by LG1 strain as we observed a redox reaction between Mn oxides and LBB reagent evidenced by the development of blue color in reaction medium. Mn oxides are among the strongest absorbents and have high oxidative power in environmental systems [35].

Proteins upregulated in the absence of Mn (II) included structural proteins and enzymes essential for the growth and survival of the bacteria. Only six proteins were over expressed in the presence of Mn, most of them related to cell membrane. The small number of differentially expressed proteins in this condition may be attributed to the fact that the LG1 is adapted to high levels of Mn (II), as was shown in our previous study [34]. The high Mn (II) concentration may have resulted in the reduction of some cellular activities and induced the expression of proteins essentially involved in cell survival under stress conditions and related to the major cell barrier, the membrane. According to Bruins et al. (2000), bacteria resist high levels of metals by modifying their cell surface properties [36]. Due to its ability to associate with metals, it has been assumed that the membrane is the site most affected by metal toxicity [37]. The outer membrane protein BamB is an important lipoprotein of the outer membrane assembly mechanism β-barrel (OMPs) and is involved in the maintenance of the membrane [38, 39]. Several studies have also demonstrated the induction of an outer membrane protein in *Pseudomonas aeruginosa* under different stress conditions [40]. ATP-dependent zinc metalloprotease FtsH, a membrane protein required for the degradation of subunits of protein complexes that are not adequately associated, contributes to the quality control of membrane proteins and to cell growth [41, 42]. D-amino acid dehydrogenase (DAD), an enzyme also bound to the membrane, catalyzes dehydrogenation reactions and subsequently produces protons and electrons that are transferred to the cytochromes of the respiratory chain [43, 44]. Enzymes in the electron transport chain can oxidize or reduce metals, a process that may lead to mineral formation in the periplasm or the cytoplasm [45]. Since was found these overexpressed protein in presence of Mn (II) have a role in the maintenance of cell membrane integrity, we suggest the high Mn (II) tolerance of LG1 strain is due to upregulation of these proteins.

In this study, the shotgun approach enabled to elucidate the first total proteome of the Mn (II)-oxidizing *S. marcescens* LG1 strain, both in the absence and presence of Mn (II). Identification of the proteins involved provided novel insights into the probable molecular mechanisms underlying the tolerance to, removal of, and protection against the toxic effects of excess Mn (II) in the LG1 strain. The findings reiterate the great potential of LG1 strain in the bioremediation of environments contaminated with Mn (II).

## Conclusion

The identification of expressed proteins in the present work provides a better physiological understanding of the inter-relationships between Mn (II)-bacteria and confirms the presence of the multicopper oxidase enzyme associated with Mn (II) removal in the *S. marcescens* LG1 strain. The present findings highlight the biotechnological value of this species; further, the list of expressed proteins identified by proteomic analysis may be used as a tool in future experiments to validate these findings.

## Methods

### Bacterial sample

The bacterial strain used was LG1 and it was confirmed as *S. marcescens* (99% similarity) by sequencing the 16S rDNA region in a previous study [34]. This strain was isolated from wastewater collected in a contaminated lake in Ouro Preto, Minas Gerais, and was selected for its resistance to Mn (II). LG1 strain was grown in solid Nutrient Broth (NB) medium (10 g/L peptone, 3 g/L yeast extract, 5 g/L NaCl, 15 g/L agar, pH 7.0) supplemented with 50 mg/L Mn (II) as MnSO_4_•H_2_O for seven days with incubation at 28 ± 2 °C. The colonies grown were cultivated in pure NB medium with constant stirring at 150 rpm at 30 °C and aliquots of the LG1 culture were preserved at − 80 °C for future experiments. The bacterial sample used in this study was collected according to the Brazilian guidelines.

### LG1 growth curve and removal of Mn (II)

To construct the growth curve, the LG1 strain was pre-cultured overnight in 10 mL of NB medium under the same conditions as above. Then, this pre-culture (in triplicate) was inoculated into 250 mL flasks containing 90 mL of pure NB medium and supplemented with 40 mg/L of Mn (II) (MnSO_4_ • H_2_O). The flasks were incubated for 48 h and aliquots were collected after 1, 2, 3, 4, 5, 6, 8, 10, 12, 24, 36 and 48 h to assess bacterial growth through optical density measurement at 600 nm (OD_600_) (Hitachi 2800A spectrophotometer).

The Mn (II) concentration and the standard cell count were determined for samples collected at 1, 24 and 48 h. These samples were centrifuged at 14,681×g for 15 min and the supernatants were analyzed by inductively coupled plasma optical emission spectrometry (Varian 725 ICP-OES) to evaluate Mn (II) decay in the medium. The removal rate of Mn (II) was calculated using the formula:

$$ Removal\ \left(\%\right)=\frac{\boldsymbol{A}-\boldsymbol{B}}{\boldsymbol{A}}\times 100\% $$

where **A** represents the initial concentration and **B** the final concentration of Mn (II). The presence of oxidized Mn species (Mn III/IV) in the 48 h culture was evaluated colorimetrically using leucoberbelin blue dye (LBB). The culture was centrifuged and 100 μL of the supernatant was added to 500 μL of 0.04% LBB (Sigma-Aldrich USA). After incubation, the medium was examined for changes in color.

Incubated 250 mL flasks containing pure NB medium supplemented with ampicillin (to prevent bacterial growth) served as controls.

### Shotgun bottom-up proteomics experiments

#### Protein extraction and short run in 1D gel electrophoresis

LG1 cultures (in three biological replicates) were grown in the presence and absence of Mn (II). After 48 h, cultures were centrifuged at 7168×g for 10 min. Cell pellets were washed with PBS 1x and suspended with 1 mL of extraction buffer (Tris HCl 50 mM pH 7.5, DTT 1 mM, diluted protease inhibitor 1:100) five times with 30 s - by sonication. Samples were incubated at 37 °C for 1 h, centrifuged at 20,000×g for 1 h and part of the supernatant was used to estimate the amount of proteins using the BCA method using BSA as standard (Sigma-Aldrich QuantiPro ™ BCA Assay Kit). The remaining supernatant was precipitated with acetone and TCA (1, 8: 1), incubated at − 20 °C overnight and then centrifuged at 18,000×g for 15 min. The pellet was washed with ice cold acetone and suspended with 100 μl of the same extraction buffer.

A short 1D gel electrophoresis (10% SDS PAGE) run was performed for 5 min until the proteins entered the gel forming a single band. This procedure aimed to concentrate the proteins and eliminate possible interference from secondary metabolites and pigments in the protein samples. As the *S. marcescens* strain LG1 is pigmented its pigment may disrupt the LC-MS/MS reading.

#### Protein digestion and LC-MS/MS analyses

Protein digestion and LC-MS/MS analyzes were performed according to Ruas et al. (2018) [46]. The single bands of the 1D gel were cut and decolorized in 40% methanol solution and 7% acetic acid at 37 °C. After washing, the gels were incubated with 50 mM DTT at 65 °C for 30 min and then with 100 mM iodoacetamide in the dark at 25 °C for 1 h, for reduction and alkylation of cysteine residues, respectively. The gel bands were washed with 20 mM NH_4_HCO_3_ for 20 min, three times, and dried. Subsequently, they were incubated with a sequencing grade modified trypsin solution (Promega, Madison, USA) with 40 mM NH_4_HCO_3_ for 20 min at 25 °C, then with 20 mM NH_4_HCO_3_ and proteolysis to proceed for 12 h at 37 °C. The supernatant was cold-preserved and the peptides were extracted from the gel using a solution of 0.1% trifluoroacetic acid (TFA) and 50% acetonitrile (ACN) for 20 min. The supernatant thus generated was added to the first supernatant, dried under vacuum and suspended in 0.1% formic acid.

The UHPLC separation of previously digested tryptic peptides was performed using a Dionex UltiMate® 3000 UHPLC system (Thermo Scientific, Bremen). 500 ng of peptides obtained from enzymatic digestion of the three biological replicates were previously washed with 2% ACN solution and 0.1% TFA for 3 min and then separated under gradient elution, using an Acclaim PepMap100 C18 Nano-Trap column (75 μm i.d × 2 cm, 3 μm, 100 Å, Thermo Scientific) online with an Acclaim PepMap100 C18 capillary column (75 μm i.d × 15 cm, 2 μm, 100 Å, Thermo Scientific). The peptides were separated with a multi-step gradient using a combination of solvents A (0.1% formic acid) and solvent B (80% ACN/0.1% formic acid) at 40 °C, and the gradient varied from 4 to 50% solvent B over 120 min.

The spectra were acquired using a Q-Exactive ™ mass spectrometer (Thermo Scientific), coupled to the UHPLC system through a Nanospray ion source. MS/MS spectra were obtained with a resolution of 17,500, a maximum injection time of 60 ms and an Automatic Gain Control (AGC) target value of 5e^5^ ions. After each MS/MS spectrum obtained, a posterior 30 s dynamic exclusion time was applied. The instrument operated at 1.9 kV, on positive ion mode, with a resolution was 70,000 at range of 300–1750 m/z range, a maximum injection time of 120 ms and an AGC target value of 1e^6^ ions. Up to 12 more intense precursor ions with charge state between 2 and 5 (excluding isotopes) were isolated from a 2 m/z window and fragmented by high-energy collisional dissociation (HCD) with normalized collision energy of 28–30 V. MS/MS spectra obtained exhibited a resolution of 17,500 with a maximum injection time of 60 ms and a AGC target ion count value of 5e^5^ ions [46].

### Data processing and bioinformatic analyses

Raw MS data were submitted to the database search PEAKS software version 8.5. Proteins were identified through comparison of MS/MS^2^ data against a UniProt compilation database containing 5075 sequences from *Serratia* (download at December 2017) and against a custom database of the predicted proteome of *S. marcescens*. The following parameters were used: enzyme trypsin, allowing at most two missed cleavages, and carbamidomethylation and oxidation of methionine as variable modifications. Other parameters such as maximum charge state (+ 7), isotope phosphorus tolerance (2 ppm) and minimum peak length (2) were also included and the precursor tolerance was set at 4.5 ppm.

The Label-Free Quantification (LFQ) method using the maxLFQ intensity data under the default mode was applied to determine the relative abundance of proteins [47]. The global parameters were selected to perform repeat quantifications and achieve matching between runs. The reversed sequences of all protein entries were concatenated to estimate the FDR. The FDR and the Peptide Sequence Matching (PSM) were set to 0.01, the minimal peptide length was 7 and one single peptide was required. The option of a second peptide was used to reduce the loss of co-eluting peptides. The set of proteins identified in the three biological replicates as differentially expressed in the absence and presence of Mn (II) were validated through the PEAKS software.

Protein annotation was obtained from UniProtKB and categorized using GO and Blast2GO which are based on the molecular function, biological process, and cellular component. Alignment of the protein sequences was also performed using Blastp via comparison with sequences present in the NCBI databases.

### Statistical analysis

The reproducibility achieved among the various chromatographic separations was evaluated by a correlation statistics (Spearman’s) test performed for each of the three biological replicates from control and Mn (II) samples, as described previously by Campos et al. (2017) [48]. Proteins exhibiting at least two maxLFQ data among the triplicates were considered for statistical analysis. The maxLFQ ratio (Mn (II) /control) against all *p*-values obtained for the identified proteins was used to produce a volcano plot to assess their pattern of up- or downregulation. Among these, only the ones that exhibited significance (*p* ≤ 0.05) were considered statistically different comparing control and Mn (II) samples [48].

## Abbreviations

EPS: Extracellular polymeric substances
LBB: Leucoberbelin blue dye
LPS: Lipopolysaccharide
MCO: Multicopper oxidase
Mn: Manganese
NB: Nutrient broth
